# Antegrade or Retrograde Accessory Pathway Conduction: Who Dies First?

**DOI:** 10.1016/s0972-6292(16)30505-8

**Published:** 2012-05-20

**Authors:** Claudio Hadid, Dario Di Toro, Sebatian Gallino, Carlos Labadet

**Affiliations:** Hospital Cosme Argerich, Buenos Aires, Argentina

**Keywords:** accessory pathway, anterograde conduction, retrograde conduction

## Abstract

A 36 year-old man with Wolff Parkinson White syndrome due to a left-sided accessory pathway (AP) was referred for catheter ablation. Whether abolition of antegrade and retrograde AP conduction during ablation therapy occurs simultaneously, is unclear. At the ablation procedure, radiofrequency delivery resulted in loss of preexcitation followed by a short run of orthodromic tachycardia with eccentric atrial activation, demonstrating persistence of retrograde conduction over the AP after abolition of its antegrade conduction. During continued radiofrequency delivery at the same position, the fifth non-preexcitated beat failed to conduct retrogradely and the tachycardia ended. In this case, antegrade AP conduction was abolished earlier than retrograde conduction.

## Case Presentation

A 36 year-old man with Wolff Parkinson White syndrome was referred for catheter ablation. The electrocardiogram during sinus rhythm showed positive delta wave in precordial and inferior leads and an isoelectric delta wave in lead I and aVL, suggesting a left-sided accessory pathway (AP). A normal echocardiogram ruled out any structural heart disease.

**Figure 1:**
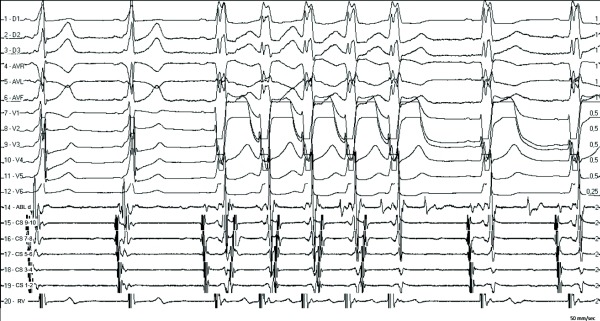
From top to bottom twelve-lead ECG and intracardiac bipolar electrograms from ablation catheter (ABL d); coronary sinus proximal (9-10) to distal (1-2) and right ventricular apex (RV).

Via right femoral vein we placed a multipolar catheter in the coronary sinus and a quadripolar catheter in right ventricular apex. A right femoral arterial puncture was made and, through a transaortic approach, a 4mm-tip ablation catheter was placed in left ventricle. We mapped the ventricular aspect of the mitral ring during sinus rhythm and in an antero-lateral position the local electrogram showed a negative delta wave-to-ventricle interval. One second after beginning delivery of radiofrequency (RF) energy, loss of preexcitation was observed (Figure 1), followed by a short run of wide QRS complex tachycardia with one-to-one ventricle-atrial (VA) relationship and eccentric atrial activation. When this tachycardia terminated, sinus rhythm with left bundle branch block (LBBB) could be seen. Five minutes after that single RF application LBBB disappeared and no preexcitation was observed. There was no recurrence of antegrade or retrograde conduction over the AP during the following 30 minutes and the ablation procedure finished without complications.

## Commentary

We present the case of a patient with Wolff Parkinson White syndrome due to a left-sided AP, successfully treated with catheter ablation. When treating AP with both antegrade and retrograde conduction, RF energy can be delivered, as in our case, during antegrade conduction (i.e. sinus rhythm with ventricular preexcitation) or during retrograde conduction (i.e. supraventricular tachycardia or continuous ventricular pacing). Completion of RF application usually results in abolition of AP, proved by absence of its antegrade and retrograde conduction. However, whether abolition of both antegrade and retrograde conduction over the AP during RF catheter ablation occurs simultaneously, is unclear.

In our case, ventricular preexcitation was abolished soon after the beginning of RF delivery. The first non-preexcitated beat showed a LBBB morphology followed by eccentric retrograde atrial activation and a short run of orthodromic supraventricular tachycardia. It can be noted that the ablation catheter recorded a continuous VA electrogram at the time of orthodromic tachycardia. During continued RF delivery at the same position, the fifth non-preexcitated beat failed to conduct retrogradely and the tachycardia terminated.

A delay in left ventricular activation and, hence, in retrograde AP penetration produced by the occurrence of LBBB led to the beginning of a short run of orthodromic tachycardia, demonstrating persistence of retrograde conduction over the AP after abolition of its antegrade conduction. This tracing shows that, in this case, antegrade AP conduction was abolished earlier than retrograde conduction.

Tai et al reported a case with abolition of antegrade AP conduction with persistence of retrograde conduction [1]. However, in that case complete AP ablation was achieved only sequentially after RF applications in closely adjacent, but anatomically separate sites. The presence of two different AP close together cannot be ruled out in that case. In our patient, as mentioned above, recordings from the ablation catheter showed continuous VA electrogram at the site of antegrade AP conduction elimination and completion of RF application without catheter movement resulted in complete abolition of VA conduction, making this possibility unlikely.

Another possibility to explain the short lasting wide QRS tachycardia is AP automaticity secondary to a rise in tissue temperature due to RF energy [2-4]. There are some arguments against this possibility. Accessory pathway automaticity usually manifests as premature atrial and ventricular activation. A ventricular impulse originating in the antero-lateral portion of the mitral ring should have a different configuration (right BBB morphology with predominantly negative QRS complex in aVL, resembling the polarity of the delta wave) than LBBB. So, if at all it is AP automaticity, it should be with conduction block over the AP to the ventricle and an eccentric atrial premature activation.Since the ablation catheter was placed in the ventricular aspect of the mitral ring (as can be deduced by the local electrogram), it is improbable that RF induced automaticity will result in atrial but not ventricular premature activation. Moreover, automatic rhythms usually exhibit a certain degree of irregularity (i.e. warm-up; cool-down) and this run of tachycardia was perfectly regular. Even so, the onset of this automaticity occurs soon after the first non-preexcitated beat, establishing a VA relationship that remains fixed until the end of the tachycardia. For all these reasons, AP automaticity is an highly unlikely explanation for the tracing.

Finally, the short lasting wide QRS tachycardia could well be catheter induced atrial tachycardia arising from the region of lateral mitral annulus occurring due to catheter movement just after elimination of preexcitation. However, position of ablation catheter on the ventricular aspect of the mitral ring argues against this possibility though it can not be ruled out completely.

The development of LBBB was considered of traumatic origin during catheter manipulation, since it spontaneously disappeared five minutes after RF delivery.

Various anatomic and histopathological factors (length and thickness of AP; atrial and ventricular insertions; serpentine course of the fibers; fibrofatty infiltration) may influence in the diverse electrophysiological characteristics of AP (i.e. bidirectional conduction with different conduction times and refractory periods of antegrade and retrograde conduction; unidirectional conduction; decremental properties). The anatomic disposition of the AP in our patient could have resulted in functional but not anatomic longitudinal dissociation of its antegrade and retrograde conduction, since complete AP ablation was achieved with a single RF application.

## References

[R1] Tai YT (1992). Successful sequential RF catheter ablation of anatomically discrete antegrade and retrograde accessory pathway conduction in the Wolff-Parkinson-White syndrome. Clin Cardiol.

[R2] Macle L (2002). Accessory pathway automaticity after RF ablation. J Cardiovasc Electrophysiol.

[R3] Tseng JH (2004). Catecholamine dependent accessory pathway automaticity. Pacing Clin Electrophysiol.

[R4] Deam AF (1995). Wide complex tachycardia due to automaticity in an accessory pathway. Pacing Clin Electrophysiol.

